# Intimate Attachment of *Escherichia coli* O157:H7 to Urinary Bladder Epithelium in the Gnotobiotic Piglet Model

**DOI:** 10.3390/microorganisms8020263

**Published:** 2020-02-15

**Authors:** Rodney A. Moxley, Tom W. Bargar, Stephen D. Kachman, Diane R. Baker, David H. Francis

**Affiliations:** 1 School of Veterinary Medicine and Biomedical Sciences, University of Nebraska-Lincoln, Lincoln, NE 68583-0905, USA; 2 Electron Microscopy Core Facility, University of Nebraska Medical Center, Omaha, NE 68198-6395, USA; tbargar@unmc.edu; 3 Department of Statistics, University of Nebraska-Lincoln, Lincoln, NE 68583-0963, USA; steve.kachman@unl.edu; 4 Department of Veterinary and Biomedical Sciences, South Dakota State University, Brookings, SD 57007, USAdavid.francis@sdstate.edu (D.H.F.)

**Keywords:** Shiga toxin-producing *E. coli*, enterohemorrhagic *E. coli*, attaching-effacing, pedestals, urinary tract infection, cystitis, pigs, gnotobiotic piglets

## Abstract

Enterohemorrhagic *Escherichia coli* (EHEC), a pathogenic subset of Shiga toxin-producing *E. coli* (STEC), is an important cause of hemorrhagic colitis and hemolytic–uremic syndrome (HUS), and a rare cause of urinary tract infections (UTIs) with associated HUS. EHEC strains attach intimately to intestinal epithelium with formation of actin pedestals (attaching-effacing (A/E) lesions); however, the mechanism of EHEC attachment to the uroepithelium is unknown. We conducted a retrospective study on archived urinary bladder specimens from gnotobiotic piglets that naturally developed cystitis associated with EHEC O157:H7 infection following oral inoculation and fecal shedding. Paraffin-embedded bladder tissues from three piglets with cystitis and immunohistochemical evidence of EHEC O157:H7 adherence to the uroepithelium were processed for and examined by transmission electron microscopy. EHEC O157:H7 bacteria were found in one of three piglets, intimately attached to pedestals on the apical surfaces of the superficial urothelium (umbrella cells). Cystitis was significantly associated with the length of survival of the piglets post-inoculation (*p* = 0.0339; estimated odds ratio = 2.6652). This is the first report of *E. coli* causing A/E-like lesions in the uroepithelium, and also evidence of the utility of the gnotobiotic piglet as a model for studies of the pathogenesis of EHEC UTIs.

## 1. Introduction

Shiga toxin-producing *Escherichia coli* (STEC) is an important cause of foodborne illness in many countries of the world [1]. STEC infections often cause hemorrhagic colitis and subsequent hemolytic–uremic syndrome (HUS) [2,3]. *E. coli* O157:H7 was the first STEC serotype recognized to cause sporadic cases and outbreaks of these illnesses [4,5,6]. Subsequently, non-O157 STEC were recognized as important causes of hemorrhagic colitis and HUS [7]. In the U.S., non-O157 outnumber O157 foodborne STEC infections, but the latter are approximately 10 times more likely to lead to a diagnosis of HUS (1% versus 11%, respectively) [8,9,10]. STEC strains isolated from human patients with hemorrhagic colitis or HUS, and those isolated from any source that carry genes that encode for virulence factors that allow them to colonize the intestine, e.g., *eae* (intimin), are known as enterohemorrhagic *E. coli* (EHEC) [11].

Although HUS commonly occurs with EHEC enteric infection, it occurs rarely in association with *E. coli* urinary tract infection (UTI). A recent systematic review of the literature found 28 individually documented cases of HUS preceded by an *E. coli* UTI in patients ranging from 2 days to 75 years of age [12]. In 19 of these cases, Shiga toxin testing of the *E. coli* isolates was done, and 15 of the isolates tested positive, i.e., they were confirmed to be EHEC. Hence, EHEC is a rare but established cause of HUS in children and adults.

Adherence to epithelial cells is a critical early step in the pathogenesis of many bacterial pathogens, including *E. coli* [13]. With regard to the urinary tract, the most common cause of uncomplicated infections is uropathogenic *E. coli* (UPEC), which accounts for approximately 90% of the cases [13,14,15]. Numerous studies have addressed the mechanisms of adherence of UPEC to the urothelium, and these findings, among other facets of the pathogenesis, have been summarized in many different reviews [13,14,16,17,18,19,20,21]. Although multiple bacterial virulence and host factors are involved, UPEC are thought to adhere to the superficial uroepithelial (umbrella) cells primarily via several different fimbria and other cell-surface adhesins, and subsequently invade these cells with the establishment of intracellular bacterial communities [13,14,15,16,17,18,19,20,21,22].

Although much is known about the pathogenesis of UPEC adherence and colonization of the uroepithelium, and also the pathogenesis of EHEC adherence to intestinal epithelium with the formation of attaching-effacing (A/E) lesions [23,24], the mechanism of adherence of EHEC to the uroepithelium is unknown. In a previous study, we reported the finding of cystitis in gnotobiotic piglets inoculated with EHEC O157:H7 strains [25]. Adherence of EHEC O157:H7 bacterial cells to the uroepithelium of the bladder as seen by light microscopy was reported in that publication [25]. In order to further investigate adherence, we conducted a retrospective study utilizing transmission electron microscopy (TEM) on blocks of formalin-fixed bladder tissues from these same piglets. EHEC O157:H7 intimately adhered to pedestals on the apical surfaces of the superficial urothelium in a manner similar to classical A/E lesions in the intestine. We also retrospectively conducted statistical analyses on clinical data of the piglets and found that the incidence of cystitis was significantly associated with the length of survival post-inoculation (PI).

## 2. Materials and Methods

### 2.1. Bacterial Strains and Gnotobiotic Piglet Studies

The bacterial strains, inoculum preparation, gnotobiotic piglet challenge studies, histological examinations, Vero cell cytotoxicity assays, and immunohistochemistries were previously described [25]. Animal experiments were approved by the South Dakota State University, Institutional Animal Care and Use Committee. All strains were EHEC O157:H7 and positive for *stx*_1_, *stx*_2_ and *eae*. The strains included EDL933 as a positive control, and 10 different strains each that were of human or bovine origin. Gnotobiotic piglets were orally inoculated 24 to 30 h after birth with ~3 × 10^9^ CFU, and observed for clinical signs of illness for a maximum of 8 d PI (Table 1). Ten piglets were inoculated with EDL933 and 5 per strain inoculated with either a human- or bovine-origin strain [25]. One litter containing control piglets that did not die or become moribund within 8 d after challenge with EDL933 was not included in the comparison of the virulence of human- and bovine-origin strains in the published study, but since urinary bladders had been collected from piglets in this litter, they were examined for cystitis [25]. With inclusion of this litter, the study included 126 piglets, with 105 having the bladder examined histologically. Individual piglets with cystitis, and their corresponding litters of origin and inoculum strains, which is information that had not been listed in the previous publication [25], is shown in Table 1.

### 2.2. Transmission Electron Microscopy

Three piglets with cystitis that had the greatest number of bacteria adherent to the mucosal epithelium visible in tissue sections (11090, 15627, and 15637) [25] (Table 1) were selected for TEM. Areas corresponding to sites of bacterial adherence were dissected from paraffin blocks, 1 to 2 mm^2^ in size. Samples were deparaffinized at 65 °C for 2 h to remove the majority of the paraffin. Following heat treatment, the pieces were passed through 4 exchanges of 100% xylene, soaking for 1 h during each exchange to remove any remaining paraffin. Samples were then placed in 2% glutaraldehyde, 2% paraformaldehyde in 0.1 M Sorensen’s phosphate buffer (SPB), pH 7.2 and stored overnight at 4 °C. The following day, samples were washed 3 times in 0.1 M SPB, soaking for 15 min in each wash. After washing, samples were post-fixed in 1% osmium tetroxide in water for 1 h, and then washed in SPB 3 times, soaking 15 min in each wash. Samples were dehydrated through a graded ethanol series 50%, 70%, 90%, 95%, 100% x 3, and 100% propylene oxide x 3, at 15 min per step. Samples were placed in a 1:1 mixture of 100% propylene oxide and Araldite 502 embedding medium (Electron Microscopy Sciences, Hatfield, PA, USA) and left overnight in a fume hood. The following day, samples were soaked in fresh Araldite 502 for 4 h, followed by final embedding in a flat silicon embedding mold in fresh Araldite 502, and placed in an oven set at 65 °C overnight for polymerization of the blocks. Sections 1 μm thick were cut from the polymerized blocks using a Diatome diamond knife (Diatome USA, Electron Microscopy Sciences) on a UC6 Ultramicrotome (Leica Microsystems, Wetzlar, Germany), then stained with toluidine blue and examined by light microscopy to locate areas of bacterial adherence to the mucosal surface. If bacteria were seen, thin sections, 60–90 nm in thickness, were then cut using the same ultramicrotome and diamond knife, and placed on 200-mesh copper grids. Sections on grids were stained with 1% uranyl acetate for 5 min, washed in water, and stained with Reynolds lead citrate for 5 min and washed. Sections were then examined on a Philips 410 transmission electron microscope operated at 80 Kv (ThermoFisher FEI, Hillsboro, OR). Images were acquired with an AMT digital camera system (AMT, Danvers, MA, USA).

### 2.3. Statistical Analyses

Cystitis was analyzed using logistic regression models. Each of the models included litter as a blocking factor. Models looking at the effect of PI days of survival included days of survival as a covariate, and models looking at the effect of bacterial strain origin (control, human or bovine) included origin as a classification factor. All analyses were carried out using the SAS^®^ software’s GLIMMIX procedure.

## 3. Results and Discussion

As noted previously, 14 of 126 (13.3%) piglets orally inoculated with EHEC O157:H7 strains developed mild to moderate purulent cystitis within 8 d PI [25] (Table 1). Further, 8 of 14 piglets with cystitis had coccobacillary bacteria attached to the apical surfaces of superficial urothelial cells, and in all eight piglets, adherent bacteria stained positive immunohistochemically for *E. coli* O157 antigen [25] (Table 1).

**Figure 1:**
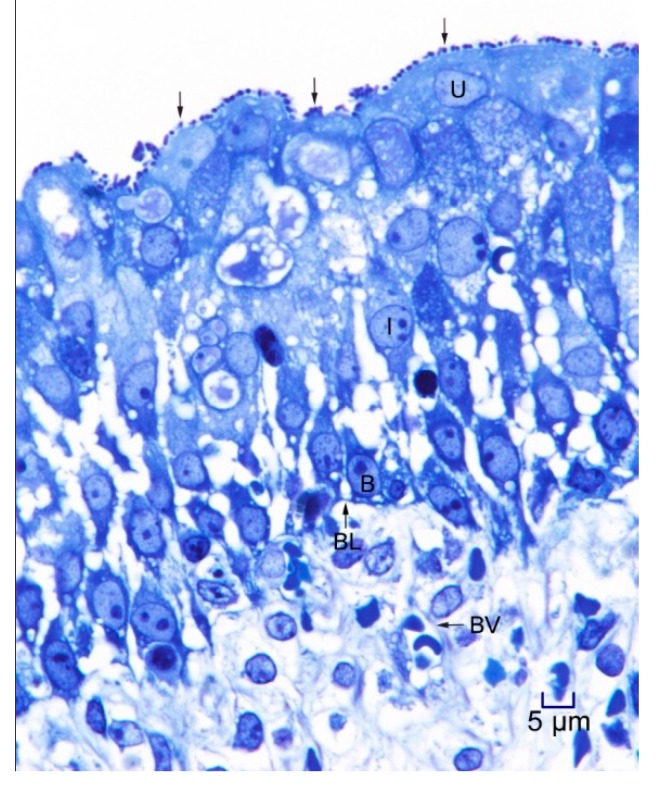
Light photomicrograph of toluidine blue-stained, 1-μm thick, Araldite section of urinary bladder of piglet 15627, 8 days post-inoculation (PI) with bovine-origin strain 2891. EHEC O157:H7 bacterial cells (arrows) are diffusely attached to the apical surfaces of superficial uroepithelial (umbrella; U) cells. The section includes all layers of the mucosa, with U cells, intermediate cells (I) and basal cells (B) present, as has been described in pigs [26,27], humans [28], and other species [29]. Basal lamina (BL) and submucosa with structures such as blood vessels (BV) are also seen in the section. Photomicrographs stained with hematoxylin and eosin (H&E) showing purulent cystitis, and positive immunohistochemical staining for *E. coli* O157 antigen of a larger sample of the same piglet specimen were shown in the previous publication [25]. Bar = 5 μm.

**Figure 2:**
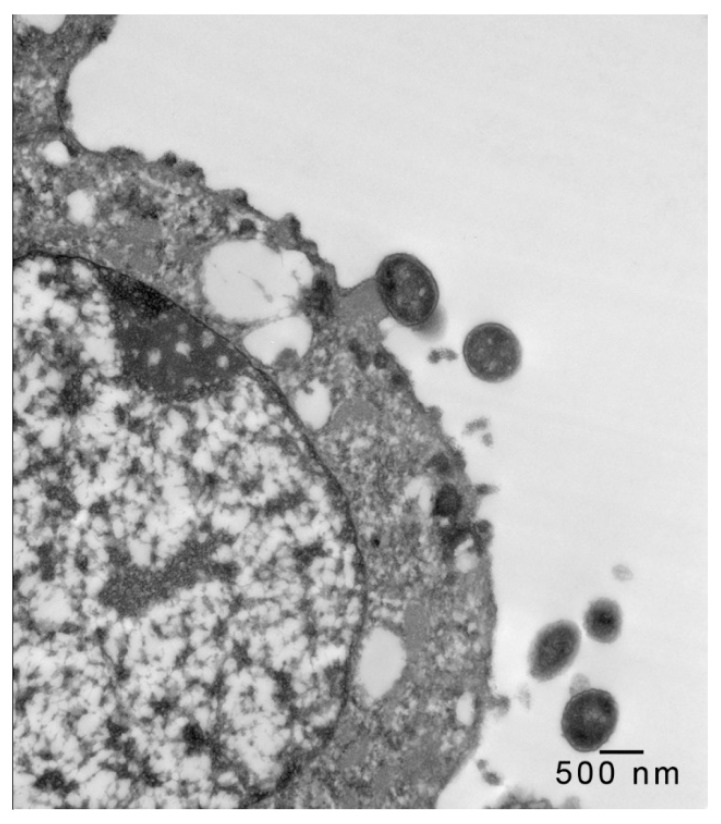
Transmission electron photomicrograph of a thin section of a superficial epithelial (umbrella) cell of the urinary bladder of piglet 15627, 8 days PI with bovine-origin strain 2891. Five bacteria are seen in the section, with one attached to an actin pedestal near the center of the figure. Bar = 500 nm.

**Figure 3:**
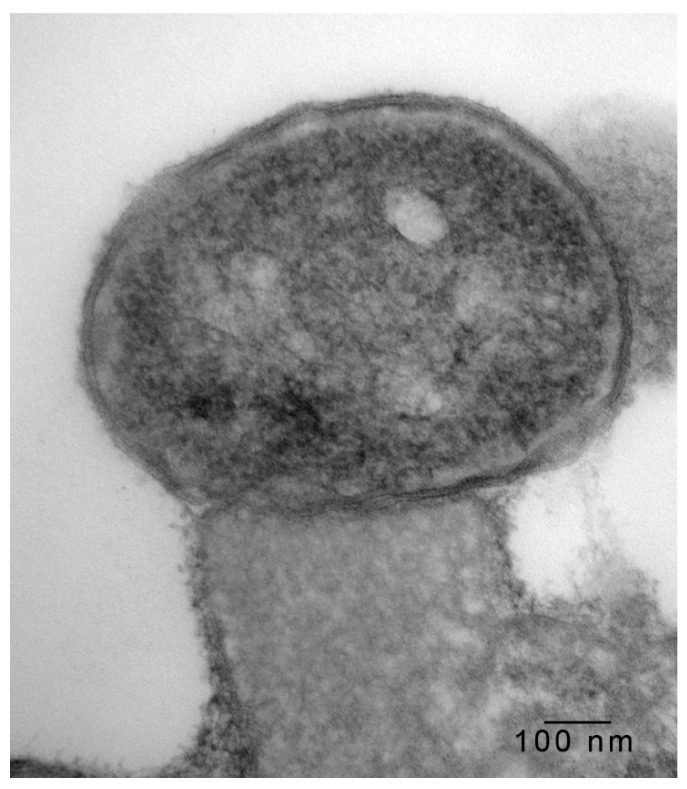
Transmission electron photomicrograph of a thin section of a superficial epithelial (umbrella) cell of the urinary bladder of piglet 15627, 8 days PI with bovine-origin strain 2891. High magnification of the bacterium intimately attached to an actin pedestal in the previous figure. Bar = 100 nm.

**Figure 4:**
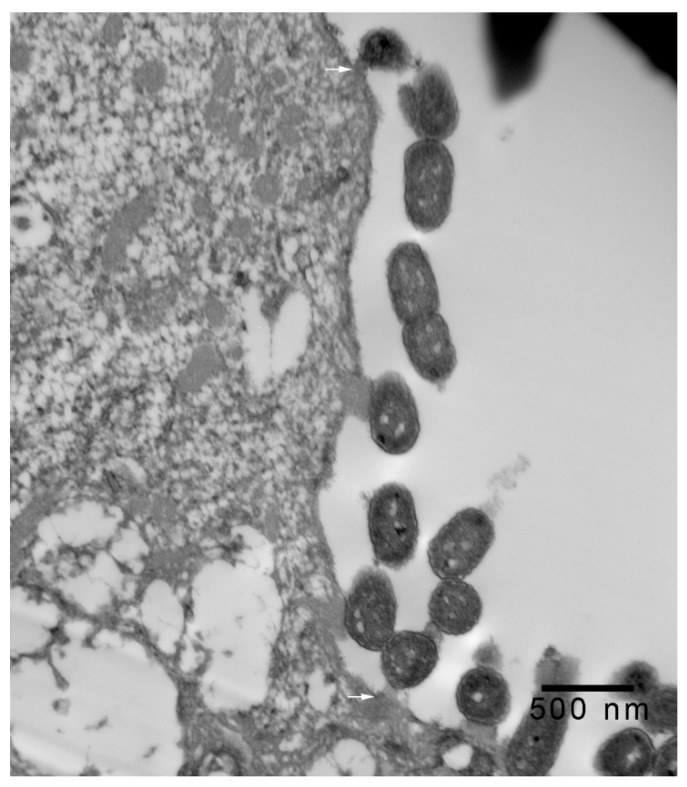
Transmission electron photomicrograph of a thin section of a superficial epithelial (umbrella) cell of the urinary bladder of piglet 15627, 8 days PI with bovine-origin strain 2891. This field shows more extensive colonization, with bacteria attached to pedestals and apparent early attachment to microplicae (arrows). Bar = 500 nm.

In 1-μm thick Araldite sections of urinary bladder prepared from tissues originally contained within the paraffin blocks [25], bacteria were detected only in piglet 15627 (Figure 1). In this piglet, in which bladder sections were oriented such that all three cell layers (basal, intermediate and superficial or umbrella) of the urothelium [26,27,28,29] were visible, bacteria, corresponding to bovine-origin strain 2891, were diffusely adherent to the apical surfaces of umbrella cells. By TEM, bacterial cells were found intimately attached to pedestals (Figure 2, Figure 3 and Figure 4). Some bacterial cells appeared to be attached to microplicae, preceding pedestal formation (Figure 4).

Morphologically, the pedestals to which bacteria in the urinary bladder of piglet 15627 were intimately attached were consistent with actin pedestals induced by EHEC and enteropathogenic *E. coli* (EPEC) in intestinal epithelium [24]. To our knowledge, this is the first report of intimate bacterial adherence and actin pedestals in the uroepithelium in any species. Staley et al. [30] first reported these lesions in 1969, describing them as attachment and microvillous exfoliation in ileal enterocytes of newborn, cesarean-derived piglets intragastrically inoculated with an *E. coli* strain belonging to a classical EPEC serotype, O55:H7. Takeuchi et al. [31] later described these lesions as occurring in rabbits inoculated with RDEC-1, a rabbit-origin O15:NM *E. coli* later classified as an EPEC [23]. Soon thereafter, the lesions were recognized in human infants with EPEC infection [32,33]. Moon et al. coined the term “attaching and effacing” to describe intimate attachment and effacement of microvilli in the intestinal epithelium of piglets and rabbits by EPEC [23]. Knutton et al. [34] first determined that the electron-dense material underlying the bacteria within the pedestals was filamentous actin. Over the past 50 years since the initial report by Staley et al. [30], numerous studies, many at the molecular level, have elucidated key bacterial and host factors involved in the pathogenesis of intimate attachment and pedestal formation [29]. 

The urinary tracts of the piglets in our study were infected naturally following exposure to the respective *E. coli* O157:H7 inoculum strains through fecal shedding and environmental contact (i.e., feed bowls and surfaces within the isolator units). Similarly, in humans, UTIs occur naturally most often through exposure to fecal microbiota [17,20,35]. Hence, we hypothesized that piglets that survived longer PI would have a higher incidence of cystitis since they would have had longer exposure to fecal and environmental bacteria in the isolator units. Indeed, the incidence of cystitis was significantly associated with the length of survival of the piglets PI (*p* = 0.0339; estimated odds ratio = 2.6652), but not the origin (control, human, or bovine) of the strain (*p* = 0.4435). In humans, UTIs are more common in females, in part due to the greater proximity of the urethral opening to the rectum and the decreased length of the urethra [17,20,35]. The same anatomical predisposition is true for females of other mammalian species, including pigs. Unfortunately, the sex of the piglets in our study had not been recorded [25]; hence, we were not able to address sex as a risk factor.

We are aware of only one other study in the literature that had addressed EHEC bacterial adherence to urothelium [36]. This was an in vitro study involving T24 human transitional cell carcinoma cells. EHEC O157:H7 bacteria were found to invade T24 cells, but there was no report of intimate adherence or pedestal formation [36]. 

The urothelium of the pig shares many features structurally and functionally with that of humans [26,27,28,37]. Since EHEC is an established, albeit rare, cause of UTIs in humans, the gnotobiotic piglet, which we have herein shown to be susceptible to spontaneous UTI following enteric infection with EHEC, may be a useful UTI model for further studies. Research questions addressed by the model could include, e.g., those aimed at the identification of fimbria or other EHEC adhesins important for uroepithelial adherence and colonization, or the testing of preventive or treatment strategies.

## Figures and Tables

**Table 1:** Distribution of piglets with cystitis in the study.

Group ^a^	Strain ^b^	Number of Piglets Inoculated	Number of Piglets with Urinary Bladder Examined	Number of Piglets with Cystitis	Number of Days Post-Inoculation Tissues Examined	Identification Numbers of Litters with Piglets Having Cystitis	Identification Numbers of Piglets with Cystitis
Control	EDL933	13	13	0	NA ^c^	NA	NA
Human	3234-86	5	2	0	NA	NA	NA
	B8763	5	4	1	5	2	2818 ^d^
	A7785	5	5	0	NA	NA	NA
	C9490	8	8	1	8	8	15637 ^d^
	B1189	5	3	0	NA	NA	NA
	C509	5	2	0	NA	NA	NA
	C6183	6	5	1	8	5	9875
	C8779	8	7	0	NA	NA	NA
	C7927	5	3	0	NA	NA	NA
	C4193	5	4	1	8	9	16506
Bovine	2890	5	4	0	NA	NA	NA
	2893	5	5	1	8	1	27 ^d^
	2903	5	5	1	8	2	2825^d^
	2909	8	5	1	8	10	4099^d^
	3032	5	4	0	NA	NA	NA
	2922	5	5	3	8	4, 9	8581 ^d^, 8582, 16507 ^e^
	2918	5	5	1	8	7	12580
	2939	5	5	1	6	5	9877
	2891	8	7	2	8	6, 8	11090 ^d^, 15627 ^df^
	2977	5	4	0	NA	NA	NA
Total		126	105	14			

^a^ EHEC O157:H7 strains were grouped into control (EDL933), human-origin, or bovine-origin. ^b^ All strains were EHEC O157:H7 and PCR-positive for *eae*, *stx*_1_ and *stx*_2_ as described in Baker et al. [25]. ^c^ NA, not applicable. ^d^ EHEC O157:H7 bacterial adherence to superficial epithelium in urinary bladder, detected histologically. ^e^ Piglets 8581 and 8582 originated from Litter 4, and piglet 16507 from Litter 9. ^f^ Piglets 11090 and 15627 originated from Litters 6 and 8, respectively.
