# Correction: Moxley, R.A., et al. Intimate Attachment of *Escherichia coli* O157:H7 to Urinary Bladder Epithelium in the Gnotobiotic Piglet Model. *Microorganisms* 2020, *8*, 263

**DOI:** 10.3390/microorganisms8122016

**Published:** 2020-12-17

**Authors:** Rodney A. Moxley, Tom W. Bargar, Stephen D. Kachman, Diane R. Baker, David H. Francis

**Affiliations:** 1 School of Veterinary Medicine and Biomedical Sciences, University of Nebraska-Lincoln, Lincoln, NE 68583-0905, USA; 2 Electron Microscopy Core Facility, University of Nebraska Medical Center, Omaha, NE 68198-6395, USA; tbargar@unmc.edu; 3 Department of Statistics, University of Nebraska-Lincoln, Lincoln, NE 68583-0963, USA; steve.kachman@unl.edu; 4 Department of Veterinary and Biomedical Sciences, South Dakota State University, Brookings, SD 57007, USA; dbaker@itctel.com (D.R.B.); david.francis@sdstate.edu (D.H.F.)

The authors wish to make the following corrections to this paper [1]:

On page 2, the sentence that reads, “Hence, EHEC is a rare but established cause of HUS in children and adults.” should read, “Hence, EHEC is a rare but established cause of UTI-associated HUS in children and adults.” On page 4, the sentence that reads, “As noted previously, 14 of 126 (13.3%) piglets orally inoculated with EHEC O157:H7 strains developed mild to moderate purulent cystitis within 8 d PI [25] (Table 1).” should read, “As noted previously, 14 of 105 (13.3%) piglets orally inoculated with EHEC O157:H7 strains in which the urinary bladder was examined developed mild to moderate purulent cystitis within 8 d PI [25] (Table 1)”.

The authors would like to apologize for any inconvenience caused to the readers by these changes.
